# Rastreamento para Hipercolesterolemia Familiar em Pequenos Municípios: A Experiência do Programa HipercolBrasil em 11 Municípios Brasileiros

**DOI:** 10.36660/abc.20201371

**Published:** 2022-01-11

**Authors:** Cinthia Elim Jannes, Júnea Paolucci Paiva Silvino, Pãmela Rodrigues de Souza Silva, Isabella Ramos Lima, Mauricio Teruo Tada, Theo Gremen Mimary Oliveira, Raul D. Santos, José Eduardo Krieger, Alexandre da Costa Pereira

**Affiliations:** 1 Universidade de São Paulo Instituto do Coração Laboratório de Genética e Cardiologia Molecular São Paulo SP Brasil Universidade de São Paulo Instituto do Coração - Laboratório de Genética e Cardiologia Molecular, São Paulo, SP – Brasil; 2 Universidade Federal de Minas Gerais Faculdade de Medicina Belo Horizonte MG Brasil Universidade Federal de Minas Gerais - Faculdade de Medicina, Belo Horizonte, MG - Brasil; 3 Universidade Federal de Mato Grosso Faculdade de Farmácia Cuiabá MS Brasil Universidade Federal de Mato Grosso - Faculdade de Farmácia, Cuiabá, MS - Brasil; 4 Universidade de São Paulo Faculdade de Medicina Instituto do Coração São Paulo SP Brasil Universidade de São Paulo Faculdade de Medicina - Instituto do Coração, São Paulo, SP – Brasil; 5 Hospital Israelita Albert Einstein Ringgold São Paulo SP Brasil Hospital Israelita Albert Einstein Ringgold, São Paulo, SP – Brasil

**Keywords:** Hipercolesterolemia Familiar, Testes Genéticos, Doenças Cardiovasculares

## Abstract

**Fundamento:**

A hipercolesterolemia familiar (HF) é uma doença genética dominante que se caracteriza por níveis sanguíneos elevados de colesterol de lipoproteína de baixa densidade (LDL-C), e está associada à ocorrência de doença cardiovascular precoce. No Brasil, o HipercolBrasil, que é atualmente o maior programa de rastreamento em cascata para HF, já identificou mais de 2.000 indivíduos com variantes genéticas causadoras de HF. A abordagem padrão baseia-se no rastreamento em cascata de casos índices referidos, indivíduos com hipercolesterolemia e suspeita clínica de HF.

**Objetivos:**

Realizar rastreamento direcionado de 11 pequenos municípios brasileiros com suspeita de alta prevalência de indivíduos com HF.

**Métodos:**

A seleção dos municípios ocorreu de 3 maneiras: 1) municípios em que houve suspeita de efeito fundador (4 municípios); 2) municípios em uma região com altas taxas de infarto do miocárdio precoce, conforme descrito pelo banco de dados do Sistema Único de Saúde (2 municípios); e 3) municípios geograficamente próximos a outros municípios com alta prevalência de indivíduos com HF (5 municípios). A significância estatística foi considerada como valor p < 0,05.

**Resultados:**

Foram incluídos 105 casos índices e 409 familiares de primeiro grau. O rendimento dessa abordagem foi de 4,67 familiares por caso índice, o qual é significativamente melhor (p < 0,0001) do que a taxa geral do HipercolBrasil (1,59). Identificamos 36 CIs com variante patogênica ou provavelmente patogênica para HF e 240 familiares de primeiro grau afetados. Conclusão: Nossos dados sugerem que, uma vez detectadas, regiões geográficas específicas justificam uma abordagem direcionada para a identificação de aglomerações de indivíduos com HF.

## Introdução

Hipercolesterolemia familiar (HF) é uma doença autossômica dominante que se caracteriza clinicamente por níveis sanguíneos elevados de colesterol de lipoproteína de baixa densidade (LDL-C), e está associada à ocorrência de doença cardiovascular aterosclerótica (DCVA) precoce.^1,2^

A prevalência da HF no mundo é estimada em aproximadamente 1:250 na forma heterozigótica e 1:600.000 na forma homozigótica.^3^ Um estudo realizado pela coorte ELSA-Brasil estimou que a prevalência de indivíduos com os critérios clínicos para a HF no Brasil seria de 1:263. Considerando essas estimativas, haveria aproximadamente 760.000 pessoas com HF no Brasil.^4^

Porém, embora seja relativamente frequente, a forma heterozigótica ainda é uma doença subdiagnosticada.^5^ Para auxiliar na identificação de indivíduos com essa doença, tem sido utilizado o rastreamento genético em cascata em vários países, como a Holanda,^6^ o Reino Unido^7^ e a Espanha.^8^ Este método já foi reconhecido como sendo de custo efetivo para a identificação e a prevenção de DCVA precoce em indivíduos com a HF.^9,10^

No Brasil, o HipercolBrasil, que é atualmente o maior programa de rastreamento genético em cascata, existe desde 2012^11^ e já identificou mais de 2.000 indivíduos com variantes genéticas causadoras de HF. O programa atualmente realiza testes genéticos em qualquer indivíduo com LDL-C ≥ 230 mg/dL (caso índice [CI])^12^ e nos familiares de primeiro grau daquelas com variantes patogênicas ou provavelmente patogênicas.

Entre julho de 2017 e julho de 2019, testamos uma nova metodologia para identificar novos indivíduos com mutações genéticas para HF, direcionada a pequenos municípios com prevalência potencialmente alta de HF.

O presente estudo descreve os primeiros resultados de triagem direcionada em 11 pequenos municípios brasileiros (até 60.000 habitantes) com suspeita de alta prevalência de pessoas com HF.

## Métodos

O estudo foi realizado no Laboratório de Genética e Cardiologia Molecular do Instituto do Coração (InCor) da Faculdade de Medicina da Universidade de São Paulo, Brasil. O protocolo foi aprovado pelo Comitê de Ética Institucional (CAPPesq protocolo l00594212.0.1001.0068).

### Amostra do estudo

**Figura 1:**
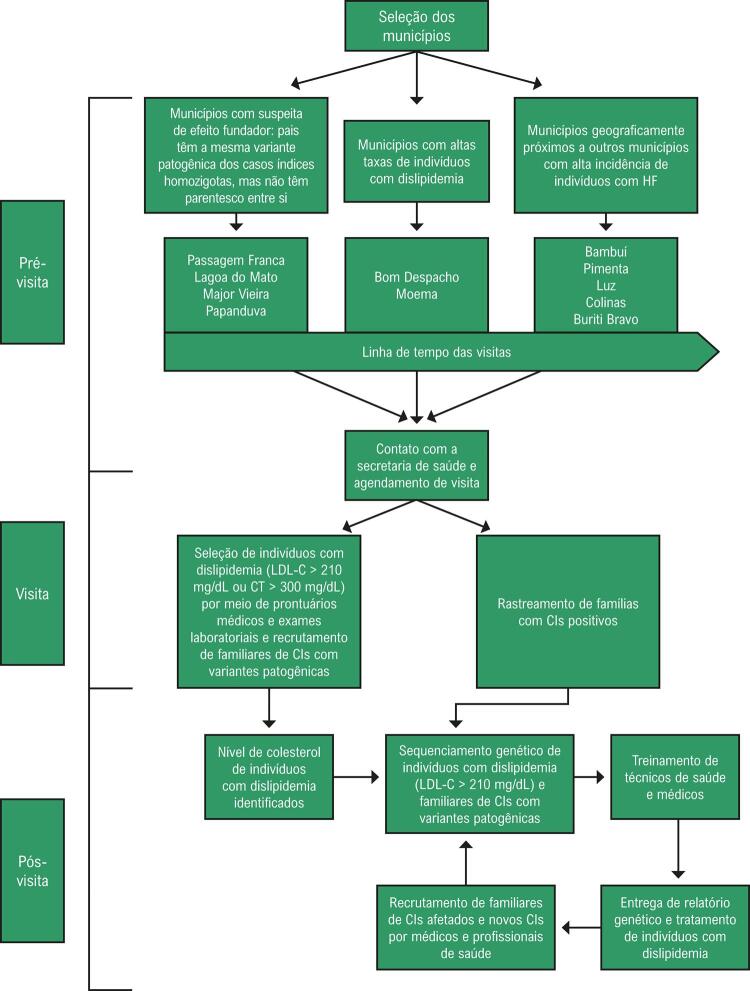
– *Metodologia para selecionar municípios, identificar CIs e familiares e treinar profissionais de saúde para continuar o rastreamento genético em cascata.*

A Figura 1 mostra os critérios de inclusão e o desenho do estudo. Cadastramos indivíduos de 11 municípios selecionados com até 60.000 habitantes em todo o território brasileiro. A seleção dos municípios ocorreu de 3 maneiras: 1) municípios em que houve suspeita de efeito fundador, ou seja, ocorrência de indivíduos homozigotos, mas sem histórico de qualquer grau de parentesco entre os pais (Major Vieira, Papanduva, Lagoa do Mato e Passagem Franca); 2) municípios em uma região com altas taxas de dislipidemia, conforme relatado por médicos locais (Bom Despacho e Moema);^13^ e 3) municípios geograficamente próximos a outros municípios com alta prevalência de indivíduos com HF (Bambuí, Pimenta, Luz, Colinas e Buriti Bravo).

### Registro de casos índices e familiares

Em todos os municípios, foi feito um contato inicial com a secretaria saúde local de para explicar o projeto e estabelecer um acordo sobre a parceria. Foi feito contato por telefone antes de visitar cada município e realizado um acordo entre ambas as partes por e-mail. Já no município, a equipe foi atendida por um agente de saúde indicado pela secretaria de saúde. Nos municípios onde havia evidências de efeito fundador e naquelas onde havia relato de alta incidência de dislipidemia, a coleta de amostras começou com familiares de CIs previamente selecionados. Nesses municípios, também ocorreu uma busca ativa para novos CIs a partir de prontuários e exames de colesterol realizados nos laboratórios de análise clínica das unidades de saúde locais. Indivíduos foram considerados CIs quando apresentavam colesterol total > 300 mg/dL e/ou LDL-C ≥ 210 mg/dL com triglicerídeos < 300 mg/dL. Nestes casos, foi coletada uma amostra de sangue para realização de uma segunda dosagem do colesterol em nosso laboratório. Aqueles com LDL-C confirmado ≥ 210 mg/dL na segunda dosagem foram selecionados para o sequenciamento genético, enquanto os indivíduos que não atingiram este valor receberam laudo com os valores de colesterol total e as frações e foram excluídos do estudo.

### Sequenciamento genético e rastreamento em cascata

Foram coletadas as amostras de sangue (10 ml de sangue periférico em tubos de EDTA) e enviadas ao Laboratório de Genética e Cardiologia Molecular do InCor/HCFMUSP para análise genética. Foi extraído o DNA genômico usando QIAamp DNA MiniKit (QIAGEN), seguindo as instruções do fabricante. Os CIs foram sequenciados por sequenciamento de próxima geração em um painel de genes incluindo os seguintes genes relacionados à dislipidemia: *LDLR, APOB, PCSK9, LDLRAP1, STAP1, LIPA, APOE, ABCG5* e *ABCG8*. Foram realizadas as análises bioinformáticas em Varstation e CLC Genomic Workbench 9.0 (QIAGEN). A amplificação multiplex de sondas dependente de ligação (MLPA) em *LDLR* foi usada para rastrear variantes de número de cópias dos CIs sem qualquer tipo de variantes do tipo *missense, nonsense* ou *frameshift* identificadas no sequenciamento de próxima geração. Foi realizado o rastreamento de familiares com sequenciamento Sanger (para mutações pontuais ou pequenos indels) ou MLPA (para variantes de número de cópias). As variantes foram classificadas de acordo com as recomendações do American College of Medical Genetics and Genomics.^14^

### Análise de dados

A análise visual da distribuição das variáveis foi realizada por meio de histogramas e foi verificada a normalidade dos dados. Para variáveis contínuas com distribuição normal, foram calculados a média e o desvio padrão. As variáveis categóricas são mostradas como frequências. As diferenças entre as frequências foram comparadas pelo teste de qui-quadrado. Foram comparadas as diferenças entre as médias com o teste t de Student não pareado ou ANOVA unilateral, se necessário. As variáveis testadas apresentaram distribuição normal e optamos pelo teste paramétrico. A significância estatística foi considerada como valor p < 0,05. As análises estatísticas foram realizadas com SPSS v19.0 (IBM).

## Resultados

Inicialmente, coletamos 230 CIs com pelo menos uma medida de colesterol que atendia aos critérios propostos (veja os Métodos). Porém, 125 deles apresentaram valores de LDL-C abaixo do ponto de corte após a segunda medição e não foi realizado sequenciamento posterior. No total, foram incluídos 105 ICs e 490 familiares na análise. A Tabela 1 mostra as características dos 11 municípios visitados, estado da federação brasileira, número de habitantes e data de cada visita. O município com o menor número de habitantes totais foi Moema com 7.028 e a maior foi Bom Despacho com 45.624, ambas no estado de Minas Gerais. Os primeiros municípios a serem visitados foram Major Vieira e Papanduva (setembro de 2017) e as últimas foram Buriti Bravo e Colinas (fevereiro de 2019).

**Tabela 1:** – Características gerais dos municípios da amostra

Município	Estado do Brasil	Habitantes totais (Censo do IBGE)	Data da visita	Número de casos esperados (1:263)^4^	Número de casos positivos identificados
Bambuí	Minas Gerais	22.709	Dez 2018	86	2
Bom Despacho	Minas Gerais	45.624	Ago 2018	173	45
Buriti Bravo	Maranhão	23.827	Fev 2019	91	0
Colinas	Maranhão	42.196	Fev 2019	160	4
Lagoa do Mato	Maranhão	10.955	Abr 2018	42	32
Luz	Minas Gerais	17.492	Dez 2018	67	6
Major Vieira	Santa Catarina	8.103	Set 2017	31	47
Moema	Minas Gerais	7.028	Ago 2018	27	36
Papanduva	Santa Catarina	18.013	Set 2017	68	48
Passagem Franca	Maranhão	17.296	Abr 2018	66	50
Pimenta	Minas Gerais	8.236	Dez 2018	31	6

*IBGE: Instituto Brasileiro de Geografia e Estatística.*

A Tabela 2 mostra o número de CIs e familiares sequenciados por região e seu genótipo em relação à presença de variantes patogênicas ou provavelmente patogênicas (positivos), sem variantes patogênicas (negativos) ou presença de uma variante de significado incerto (VSI), bem como o número de novos casos derivados de cada CI incluído.

**Tabela 2:** – CIs e familiares incluídos por região e seus genótipos para a presença de variantes genéticas de HF

Origem	CIs			Familiares			Número de familiares por CIs identificados	Número de indivíduos genotipados por município

	Negativo	Positivo	VSI	Negativo	Positivo	VSI		
Bambuí	0	1	0	0	1	0	1	2
Bom Despacho	15	11	2	34	31	3	2,4	96
Buriti Bravo	4	0	0	0	0	0	0	4
Colinas	6	1	1	1	3	0	0,5	12
Lagoa do Mato	3	2	0	25	30	0	11	60
Luz	21	4	1	0	2	0	0,08	28
Major Vieira	1	3	0	48	44	0	23	96
Moema	1	4	0	36	32	0	13,6	73
Papanduva	4	2	1	50	46	0	13,7	103
Passagem Franca	3	5	0	55	45	0	12,5	108
Pimenta	6	2	1	0	4	0	0,4	13
Total	64	35	6	249	238	3	4,7	595

*CI: caso índice; VSI: variante de significado incerto; HF: Hipercolesterolemia familiar.*

A Tabela 3 mostra os três grupos de CIs (negativo, positivo ou VSI) e seus dados clínicos e bioquímicos. No total, foram sequenciados 105 CIs, sendo encontradas variantes patogênicas ou provavelmente patogênicas em 36 (37,8%) indivíduos e VSI em 5 (5,25%). A maioria dos CIs era do sexo feminino (67,6%) e quando as características clínicas e bioquímicas foram avaliadas entre os três grupos, houve, como esperado, uma diferença estatisticamente significativa em relação ao colesterol total e LDL-C basais (não tratados), com o grupo positivo apresentando os maiores valores de colesterol total e LDL-C, 382 ± 150 mg/dL e 287 ± 148 mg/dL, respectivamente. A Tabela 4 mostra as características clínicas e bioquímicas dos familiares.

**Tabela 3:** – Características clínicas e bioquímicas de CIs negativos, positivos e alterados por VSI

	CI negativo	(64)	CI positivo	(36)	CI com VSI	(5)	valor p
Mulheres %	45 (70,3)	64	21 (58,3)	36	5 (100)	5	0,134
Homens %	19 (29,7)	64	15 (41,7)	36	-	5	
Idade (anos)	54±15	64	44±19	36	56±16	5	0,015
Uso de drogas hipolipemiantes	32 (50,0)	64	24 (66,7)	36	3 (60,0)	5	0,261
DAC precoce	2 (3,1)	64	4 (11,1)	36	-	5	0,297
Xantomas	3 (4,7)	64	3 (8,3)	36	1 (20,0)	5	0,365
Xantelasmas	4 (6,3)	64	1 (2,8)	36	-	5	0,696
Arco córneo	2 (3,1)	64	3 (8,3)	36	-	5	0,345
CT atual	279±65	62	316±107	36	302±28	5	0,102
LDL-C atual	195±56	64	234±104	36	207±35	5	0,051
CT basal	322±33	60	382±150	32	305±43	5	0,008
LDL-C basal	233±24	59	287±148	34	229±20	4	0,022

*CI: caso índice; CT: colesterol total; DAC: doença arterial coronariana; LDL-C: colesterol de lipoproteína de baixa densidade; VSI: variante de significado incerto. DAC precoce definido como evento de doença cardiovascular aterosclerótica < 55 e 60 anos de idade em homens e mulheres, respectivamente; lipídios em mg/dL; lipídios basais = não tratados.*

**Tabela 4:** – Características clínicas e bioquímicas dos familiares positivos e negativos

	Familiares negativos	N (249)	Familiares positivos	N (240)	valor p
Mulheres %	136 (54,6)	249	135 (56,3)	240	0,504
Homens %	113 (45,4)	249	105 (43,8)	240	
Idade (anos)	40±21	249	38±21	240	0,710
Uso de drogas hipolipemiantes	31 (12,4)	249	93 (38,8)	240	0,001
DAC precoce	2 (0,8)	249	9 (3,8)	240	0,034
Xantomas	6 (2,4)	249	17 (7,1)	240	0,013
Xantelasmas	11 (4,4)	249	34 (14,2)	240	0,001
Arco córneo	1 (0,4)	249	9 (3,8)	240	0,009
CT atual	198±51	114	309±86	127	0,001
LDL-C atual	124±42	192	233±75	198	0,001
CT basal	220±191	97	318±97	130	0,001
LDL-C basal	126±41	169	243±82	178	0,001

*DAC: doença arterial coronariana; LDL-C: colesterol de lipoproteína de baixa densidade; CT: colesterol total. DAC precoce definido como evento de doença cardiovascular aterosclerótica < 55 e 60 anos de idade em homens e mulheres, respectivamente; lipídios em mg/dL; lipídios basais = não tratados.*

**Figura 2:**
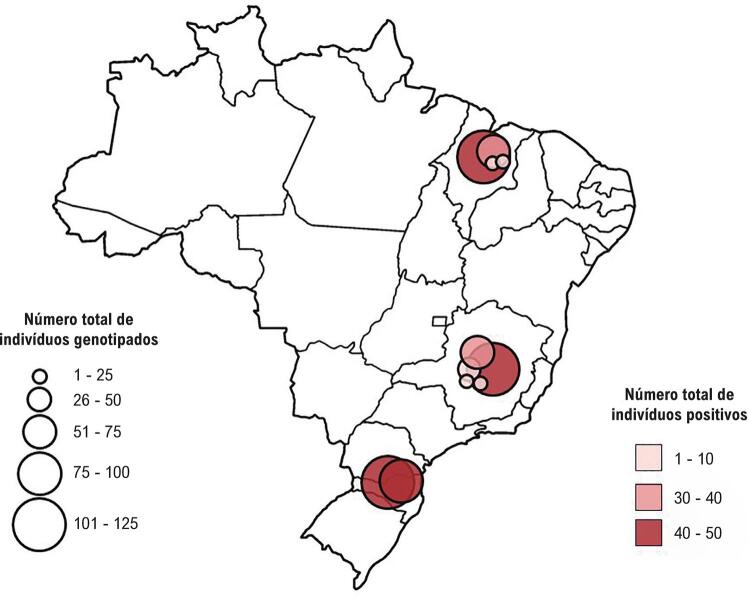
– *Distribuição geográfica dos casos, número de indivíduos genotipados e número de indivíduos com variante patogênica identificada (positivos).*

A Figura 2 mostra a distribuição geográfica dos 11 municípios localizados em 3 estados da federação brasileira, o número de casos registrados, o número de indivíduos genotipados e o número de indivíduos com uma variante patogênica.

### Estados brasileiros, de cima para baixo: Maranhão, Minas Gerais e Santa Catarina

A Tabela 5 mostra todas as variantes encontradas e o local onde foram identificadas. No total, foram identificadas 21 variantes diferentes, com 3 variantes aparecendo com mais frequência. As frequências observadas para essas 3 variantes sugerem que elas têm efeitos fundadores nessas localidades. Foram encontrados 6 pacientes homozigotos e 1 heterozigoto composto em trans.

**Tabela 5:** – Variantes patogênicas, provavelmente patogênica e VSI de HF encontradas por município

Gene	Variante	Classificação da variantes	Bambuí	Bom Despacho	Luz	Pimenta	Moema	Buriti Bravo	Colinas	Lagoa do Mato	Passagem Franca	Major Vieira	Papanduva	Total
LDLR	Duplicação do exon 4 para 8 (b)	Patogênica	0	0	0	0	0	0	0	0	0	45^b^	41	86
LDLR	Duplicação do promoter para o exon 6	Patogênica	0	0	0	0	0	1	4	29	49^a^	0	0	83
LDLR	p.Asp224Asn	Patogênica	0	39	4	0	34	0	0	0	0	0	0	77
LDLR	p.Cys222*	Patogênica	0	0	0	0	0	0	0	0	0	0	5	5
LDLR	c.1359-1G >C	Patogênica	0	0	0	5	0	0	0	0	0	0	0	5
LDLR	p.Gly592Glu	Patogênica	0	0	0	0	0	0	0	0	0	2	0	2
LDLR	p.Ala771Val	Patogênica	0	0	1	0	0	0	0	0	0	0	0	1
LDLR	p.Pro699Leu	Patogênica	0	0	1	0	0	0	0	0	0	0	0	1
LDLR	p.Asp601His	Provavelmente patogênica	2	0	0	0	2	0	0	0	0	0	0	4
LDLR	p.Cys34Arg	Provavelmente patogênica	0	1	0	0	0	0	0	0	0	0	0	1
LDLR	p.Arg257Trp	Provavelmente patogênica	0	0	0	0	0	0	0	0	0	0	1	1
LDLR	p.Ser854Gly	Provavelmente patogênica	0	2	0	0	0	0	0	0	0	0	0	2
LDLR	c.-228G>C	VSI	0	0	0	0	0	0	1	0	0	0	0	1
LDLR	p.Ala30Gly	VSI	0	0	0	1	0	0	0	0	0	0	0	1
APOB	p.Ala2790Thr	VSI	0	0	0	0	0	0	0	0	0	0	1	1
APOB	p.Met499Val	VSI	0	1	0	0	0	0	0	0	0	0	0	1
PCSK9	p.Arg237Trp	VSI	0	4	0	0	0	0	0	0	0	0	0	4
PCSK9	p.Arg357Cys	VSI	0	0	1	0	0	0	0	0	0	0	0	1
STAP1	p.Pro176Ser	VSI	0	0	0	1	0	0	0	0	0	0	0	1
LDLR	p.Cys222*	Patogênica	0	0	0	0	0	0	0	0	0	0	1^c^	1^c^
LDLR	Duplicação do exon 4 para 8	Patogênica												
PCSK9	p.Arg215Cys	Provavelmente patogênica	0	0	0	0	0	0	0	1	0	0	0	1^c^
APOB	p.Asp2213Asn	VSI												
APOB	p.Val3290Ile	VSI												
PCSK9	p.Arg215Cys	Provavelmente patogênica	0	0	0	0	0	0	0	1	0	0	0	1^c^
APOB	p.Val3293lle	VSI												
PCSK9	p.Arg215Cys	Provavelmente patogênica	0	0	0	0	0	0	0	1	0	0	0	1^c^
APOB	p.Asp2213Asn	VSI												

*2 homozigotas (b) 4 homozigotas (c) heterozigota composta em trans. VSI: variante de significado incerto; HF: Hipercolesterolemia familiar.*

## Discussão

O presente estudo descreve os resultados da implementação de um sistema de rastreamento em cascata para HF em 11 pequenos municípios brasileiros.

Apesar dos benefícios de custo conhecidos do rastreamento em cascata para HF, a implementação mundial tem sido abaixo do ideal. Diversas barreiras locais e obstáculos à implementação devem ser identificados e superados. A implementação do rastreamento em cascata em pequenas localidades, por exemplo, tem sido maiormente desconsiderado. Esse desafio é maior em países de dimensões continentais, como o Brasil, onde, além das enormes distâncias geográficas, existem desigualdades no acesso aos serviços de saúde. Descrevemos a experiência do HipercolBrasil que realizou rastreamento em cascata abrangente em pequenos municípios brasileiros. Neste novo modelo, o rastreamento genético em cascata foi realizado em municípios que apresentavam evidências de maior prevalência de HF devido ao achado prévio de indivíduos com o fenótipo homozigoto no mesmo município, ou porque essas regiões relataram elevada frequência de infarto do miocárdio.

Os municípios que apresentaram evidências de efeito fundador foram os que apresentaram maior identificação de indivíduos afetados por cada CI analisado (em ordem decrescente Major Vieira, Papanduva, Lagoa do Mato e Passagem Franca). Nestas cidades, começamos com indivíduos homozigotos cujos pais não tinham parentesco e nasceram em regiões geográficas diferentes. Obviamente, sempre que essa situação for sinalizada por um programa de rastreamento em cascata, ela merece a implantação de uma abordagem que abranja todo o município, pois os custos-benefícios deste cenário são os mais vantajosos. A implementação da cascata genética em municípios de pequeno porte mostrou-se mais eficiente quando comparada à cascata genética realizada pelo HipercolBrasil^11^ considerando que as taxas de familiares por CI foram de 4,7 e 1,6, respectivamente (p < 0,0001).

É importante notar que a taxa de familiares testados por CI também foi maior em municípios com suspeita de efeito fundador. Isso provavelmente ocorreu porque esses municípios possuíam um número pequeno de habitantes e a maioria dos familiares tinha algum grau de relação familiar. Isso não ocorreu em Bom Despacho, que é um município consideravelmente maior que os demais (45.624 habitantes) e, embora o número de familiares coletados tenha sido semelhante ao de outras cidades, houve maior número de CIs coletados (28), diminuindo a taxa de parentes/CI para 2,4. Essa situação exemplifica o equilíbrio tênue entre o tamanho do município e o sucesso da abordagem descrita.

Os municípios visitados que eram geograficamente próximos a municípios com suspeita de efeito fundador (Bambuí, Buriti Bravo, Colinas, Pimenta e Luz) apresentaram baixa captação de CIs e, consequentemente, baixo número de familiares identificados. Isso sugere que a concentração de esforços no município selecionado, ao invés de estender a abordagem para cidades próximas, deve ser priorizada e a captura de casos potenciais próximos deve ser deixada para o mecanismo usual de rastreamento em cascata.

## Conclusão

Rastreamento em cascata em pequenos municípios (menos de 60.000 habitantes) com efeito fundador mostrou-se eficaz. Porém, alguns pontos podem ser de grande importância para que o rastreamento em cascata seja eficaz, podendo ser considerados os seguintes antes de decidir quais cidades rastrear: estabelecimento de uma parceria formal e interesse explícito por parte do departamento de saúde local em receber o programa e realizar o rastreamento em cascata; disponibilidade de conjuntos de dados laboratoriais de análises clínicas para a realização de levantamento retrospectivo dos testes de colesterol; e divulgação via rádios e redes sociais sobre a doença e o programa para maior adesão dos moradores.

O presente estudo é limitado pelo número relativo de municípios avaliados considerando o tamanho continental do Brasil. No entanto, sugere que a abordagem desenhada pode ser útil para detectar indivíduos com HF. Em conclusão, nossos dados sugerem que, uma vez detectadas, regiões geográficas específicas justificam uma abordagem direcionada para a identificação de aglomerações de indivíduos com HF.

## References

[1] Goldberg AC, Hopkins PN, Toth PP, Ballantyne CM, Rader DJ, Robinson JG, et al. Familial Hypercholesterolemia: Screening, Diagnosis and Management of Pediatric and Adult Patients: Clinical Guidance from the National Lipid Association Expert Panel on Familial Hypercholesterolemia. J Clin Lipidol. 2011;5(3 Suppl):1-8. doi: 10.1016/j.jacl.2011.04.003. 10.1016/j.jacl.2011.04.003 21600525

[2] van der Graaf A, Kastelein JJP, Wiegman A. Heterozygous Familial Hypercholesterolaemia in Childhood: Cardiovascular Risk Prevention. J Inherit Metab Dis. 2009;32(6):699. doi: 10.1007/s10545-009-1165-1. 10.1007/s10545-009-1165-1 19898954

[3] Hopkins PN, Toth PP, Ballantyne CM, Rader DJ; National Lipid Association Expert Panel on Familial Hypercholesterolemia. Familial Hypercholesterolemias: Prevalence, Genetics, Diagnosis and Screening Recommendations from the National Lipid Association Expert Panel on Familial Hypercholesterolemia. J Clin Lipidol. 2011;5(3 Suppl):9-17. doi: 10.1016/j.jacl.2011.03.452. 10.1016/j.jacl.2011.03.452 21600530

[4] Harada PH, Miname MH, Benseñor IM, Santos RD, Lotufo PA. Familial Hypercholesterolemia Prevalence in an Admixed Racial Society: Sex and RACE MATter. The ELSA-Brasil. Atherosclerosis. 2018;277:273-7. doi: 10.1016/j.atherosclerosis.2018.08.021. 10.1016/j.atherosclerosis.2018.08.021 30270058

[5] Nordestgaard BG, Chapman MJ, Humphries SE, Ginsberg HN, Masana L, Descamps OS, et al. Familial Hypercholesterolaemia is Underdiagnosed and Undertreated in the General Population: Guidance for Clinicians to Prevent Coronary Heart Disease: Consensus Statement of the European Atherosclerosis Society. Eur Heart J. 2013;34(45):3478-90. doi: 10.1093/eurheartj/eht273. 10.1093/eurheartj/eht273 PMC3844152 23956253

[6] Umans-Eckenhausen MA, Defesche JC, Sijbrands EJ, Scheerder RL, Kastelein JJ. Review of First 5 Years of Screening for Familial Hypercholesterolaemia in the Netherlands. Lancet. 2001;357(9251):165-8. doi: 10.1016/S0140-6736(00)03587-X. 10.1016/S0140-6736(00)03587-X 11213091

[7] Hadfield SG, Horara S, Starr BJ, Yazdgerdi S, Marks D, Bhatnagar D, et al. Family Tracing to Identify Patients with Familial Hypercholesterolaemia: The Second Audit of the Department of Health Familial Hypercholesterolaemia Cascade Testing Project. Ann Clin Biochem. 2009;46(Pt 1):24-32. doi: 10.1258/acb.2008.008094. 10.1258/acb.2008.008094 19028807

[8] Mozas P, Castillo S, Tejedor D, Reyes G, Alonso R, Franco M, et al. Molecular Characterization of Familial Hypercholesterolemia in Spain: Identification of 39 Novel and 77 Recurrent Mutations in LDLR. Hum Mutat. 2004;24(2):187. doi: 10.1002/humu.9264. 10.1002/humu.9264 15241806

[9] Sperlongano S, Gragnano F, Natale F, D’Erasmo L, Concilio C, Cesaro A, et al. Lomitapide in Homozygous Familial Hypercholesterolemia: Cardiology Perspective From a Single-Center Experience. J Cardiovasc Med. 2018;19(3):83-90. doi: 10.2459/JCM.0000000000000620. 10.2459/JCM.0000000000000620 29389816

[10] 0. Lázaro P, Isla LP, Watts GF, Alonso R, Norman R, Muñiz O, et al. Cost-Effectiveness of a Cascade Screening Program for the Early Detection of Familial Hypercholesterolemia. J Clin Lipidol. 2017;11(1):260-71. doi: 10.1016/j.jacl.2017.01.002. 10.1016/j.jacl.2017.01.002 28391894

[11] Jannes CE, Santos RD, Silva PRS, Turolla L, Gagliardi ACM, Marsiglia JDC, et al. Familial Hypercholesterolemia in Brazil: Cascade Screening Program, Clinical and Genetic Aspects. Atherosclerosis. 2015;238(1):101-7. doi: 10.1016/j.atherosclerosis.2014.11.009 10.1016/j.atherosclerosis.2014.11.009 25461735

[12] Santos RD, Bourbon M, Alonso R, Cuevas A, Vasques-Cardenas NA, Pereira AC, et al. Clinical and Molecular Aspects of Familial Hypercholesterolemia in Ibero-American Countries. J Clin Lipidol. 2017;11(1):160-6. doi: 10.1016/j.jacl.2016.11.004. 10.1016/j.jacl.2016.11.004 28391882

[13] Silvino JPP, Jannes CE, Tada MT, Lima IR, Silva IFO, Pereira AC, et al. Cascade Screening and Genetic Diagnosis of Familial Hypercholesterolemia in Clusters of the Southeastern Region from Brazil. Mol Biol Rep. 2020;47(12):9279-88. doi: 10.1007/s11033-020-06014-0. 10.1007/s11033-020-06014-0 33231818

[14] Richards S, Aziz N, Bale S, Bick D, Das S, Gastier-Foster J, et al. Standards and Guidelines for the Interpretation of Sequence Variants: A Joint Consensus Recommendation of the American College of Medical Genetics and Genomics and the Association for Molecular Pathology. Genet Med. 2015;17(5):405-24. doi: 10.1038/gim.2015.30. 10.1038/gim.2015.30 PMC4544753 25741868

