# Supplementary material for: Rastreamento para Hipercolesterolemia Familiar em Pequenos Municípios: A Experiência do Programa HipercolBrasil em 11 Municípios Brasileiros
Source: Arq Bras Cardiol. 2022 Jan 11;118(4):669–77. [Article in Portuguese] doi: 10.36660/abc.20201371 (PMC9007005; doi:10.36660/abc.20201371)
Supplement: Translation

# Translation: Screening for Familial Hypercholesterolemia in Small Towns: Experience from 11 Brazilian Towns in the Hipercolbrasil Program

**Authors:** Cinthia Elim Jannes, Júnea Paolucci Paiva Silvino, Pãmela Rodrigues de Souza Silva, Isabella Ramos Lima, Mauricio Teruo Tada, Theo Gremen Mimary Oliveira, Raul D. Santos, José Eduardo Krieger, Alexandre da Costa Pereira

**Affiliations:** 1 Brasil Universidade de São Paulo Instituto do Coração - Laboratório de Genética e Cardiologia Molecular, São Paulo, SP – Brasil; 2 Brazil Universidade Federal de Minas Gerais - Faculdade de Medicina, Belo Horizonte, MG – Brazil; 3 Brazil Universidade Federal de Mato Grosso - Faculdade de Farmácia, Cuiabá, MS – Brazil; 4 Brazil Universidade de São Paulo Faculdade de Medicina - Instituto do Coração, São Paulo, SP –– Brazil; 5 Brazil Hospital Israelita Albert Einstein Ringgold, São Paulo, SP – Brazil

**Keywords:** Familial hypercholesterolemia, Genetic Testing, Cardiovascular Disease

## Abstract

**Background:**

Familial hypercholesterolemia (FH) is a genetic disease characterized by elevated serum levels of low-density lipoprotein cholesterol (LDL-C), and it is associated with the occurrence of early cardiovascular disease. In Brazil, HipercolBrasil, which is currently the largest FH cascade screening program, has already identified more than 2000 individuals with causal genetic variants for FH. The standard approach is based on cascade screening of referred index cases, individuals with hypercholesterolemia and clinical suspicion of FH.

**Objectives:**

To perform targeted screening of 11 small Brazilian cities with a suspected high prevalence of people with FH.

**Methods:**

The selection of cities occurred in 3 ways: 1) cities in which a founder effect was suspected (4 cities); 2) cities in a region with high rates of early myocardial infarction as described by the National Health System database (2 cities); and 3) cities that are geographically close to other cities with a high prevalence of individuals with FH (5 cities). Statistical significance was considered as p value < 0.05.

**Results:**

One hundred and five index cases and 409 first-degree relatives were enrolled. The yield of such approach of 4.67 relatives per index case was significantly better (p < 0.0001) than the general HipercolBrasil rate (1.59). We identified 36 IC with a pathogenic or likely pathogenic variant for FH and 240 affected first-degree relatives.

**Conclusion:**

Our data suggest that, once detected, specific geographical regions warrant a target approach for identification of clusters of individuals with FH.

## Introduction

Familial hypercholesterolemia (FH) is an autosomal dominant disease that is clinically characterized by elevated blood levels of low density lipoprotein cholesterol (LDL-C), and it is associated with the occurrence of early atherosclerotic cardiovascular disease (ASCVD).^1,2^

The prevalence of FH in the world is estimated to be approximately 1:250 in the heterozygous form and 1:600,000 in the homozygous form..A study conducted by the ELSA-Brasil cohort estimated that the prevalence of individuals with clinical criteria for FH in Brazil is 1:263. Considering these estimates, there would be approximately 760,000 people with FH in Brazil.^4^

However, although relatively frequent, the heterozygous form is still an underdiagnosed disease.^5^ To assist in the identification of individuals with this disease, cascade genetic screening has been used in several countries, such as the Netherlands,^6^ the United Kingdom,^7^ and Spain.^8^ This method has already been recognized as cost-effective for identification as well as prevention of early ASCVD in individuals with FH.^9,10^

In Brazil, HipercolBrasil, which is currently the largest cascade screening program, has existed since 2012,^11^ and it has already identified more than 2000 individuals with causal genetic variants for FH. The program currently performs genetic testing on any individual with LDL-C ≥ 230mg/dL (index-case [IC])^12^ and in first-degree relatives of those with pathogenic or likely pathogenic variants.

Between July 2017 and July 2019 we tested a new methodology for identifying new individuals with genetic alterations for FH based on the targeting of small municipalities with potentially high FH prevalence.

Here we describe the first results of targeted screening in 11 small Brazilian cities (up to 60,000 inhabitants) with a suspected high prevalence of people with FH.

## Methods

The study was conducted at the Genetics and Molecular Cardiology Laboratory of the Heart Institute (InCor), University of São Paulo Medical School, São Paulo, Brazil. The protocol received approval from the Institutional Ethics Committee (CAPPesq protocol l00594212.0.1001.0068).

### Study sample

Figure 1 shows inclusion criteria and study design. We enrolled individuals from 11 selected cities with up to 60,000 inhabitants throughout the Brazilian territory. The selection of cities occurred in 3 ways: 1) cities in which a founder effect was suspected, i.e. occurrence of homozygous individuals, but with no history of any degree of relation between parents (Major Vieira, Papanduva, Lagoa do Mato, and Passagem Franca); 2) cities in a region with high rates of dyslipidemia as reported by local physicians (Bom Despacho and Moema);^13^ and 3) cities that are geographically close to other cities with a high prevalence of individuals with FH (Bambuí, Pimenta, Luz, Colinas, and Buriti Bravo).

**Figure 1:** – Methodology for selecting cities, capturing ICs and relatives and training health care professionals to continue cascade genetic screening.

### Enrolment of index cases and relatives

In all cities, initial contact was made with the local secretary of health to explain the project and establish an agreement on the partnership. Contact was made via telephone before visiting each city, and an agreement was established by both parties via e-mail. Once in the city, the team was assisted by a health agent appointed by the health secretary. In the cities where there was evidence of a founder effect and in the ones where there were reports of high incidence of dyslipidemia, the sample collection started from family members of previously selected ICs. In these cities, there was also an active search for new ICs from medical records and cholesterol tests carried out in the clinical analysis laboratories of the local healthcare units. Individuals were considered as ICs when they had total cholesterol > 300 mg/dL and/or LDL-C ≥ 210 mg/dL with triglycerides < 300 mg/dL. In these cases, a blood sample was collected to perform a second cholesterol measurement in our laboratory. Those with a confirmed LDL-C ≥ 210 mg/dl in the second measurement were selected for genetic sequencing, while individuals who did not reach this value received a report with the values of total cholesterol and fractions and were excluded from the study.

### Genetic sequencing and cascade screening

Blood samples were collected (10 ml of peripheral blood in EDTA tubes) and sent to the Genetics and Molecular Cardiology Laboratory at InCor/HCFMUSP for genetic analysis. Genomic DNA was extracted using QIAamp DNA MiniKit (QIAGEN), following the manufacturer’s instructions. IC were sequenced by next generation sequencing in a gene panel comprising the following dyslipidemia-related genes: *LDLR, APOB, PCSK9, LDLRAP1, STAP1, LIPA, APOE, ABCG5*, and *ABCG8*. Bioinformatics analyses were performed in Varstation and CLC Genomic Workbench 9.0 (QIAGEN). Multiplex ligation-dependent probe amplification (MLPA) in *LDLR* was used to screen for copy-number variants in ICs without any missense, nonsense or frameshift variants identified in next generation sequencing. The screening of relatives was performed with Sanger sequencing (for point mutations or small indels) or MLPA (for copy-number variants). Variants were classified following the recommendations of the American College of Medical Genetics and Genomics.^14^

### Data analysis

The visual analysis of variable distribution was performed using histograms, and the normality of the data was verified. For continuous variables with normal distribution, the mean and standard deviation were calculated. Categorical variables are shown as frequencies. The differences between frequencies were compared using the chi-square test. The differences between means were compared with unpaired Student’s t test or one-way ANOVA, if necessary. The tested variables were normally distributed, and we opted for a parametric test. Statistical significance was considered as p value < 0.05. Statistical analyses were performed with SPSS v19.0 (IBM).

## Results

Initially, we collected 230 ICs with at least one cholesterol measure that met the proposed criteria (see Methods). However, 125 of them presented LDL-C values below the threshold after the second measurement and were not further sequenced. In total, 105 ICs and 490 relatives were included in the analysis. Table 1 shows characteristics of the 11 visited cities, Brazilian state, number of inhabitants, and date of each visit. The city with the lowest number of total inhabitants was Moema with 7,028, and the largest was Bom Despacho with 45,624 inhabitants, both in the state of Minas Gerais. The first cities to be visited were Major Vieira and Papanduva (September 2017) and the last were Buriti Bravo and Colinas (February 2019).

**Table 1:** – Overall characteristics of sampled municipalities

City	Brazilian state	Total inhabitants (IBGE Census)	Visit date	N of expected cases (1:263)^4^	N of positive cases identified
Bambuí	Minas Gerais	22,709	Dec 2018	86	2
Bom Despacho	Minas Gerais	45,624	Aug 2018	173	45
Buriti Bravo	Maranhão	23,827	Feb 2019	91	0
Colinas	Maranhão	42,196	Feb 2019	160	4
Lagoa do Mato	Maranhão	10,955	Apr 2018	42	32
Luz	Minas Gerais	17,492	Dec 2018	67	6
Major Vieira	Santa Catarina	8,103	Sep 2017	31	47
Moema	Minas Gerais	7,028	Aug 2018	27	36
Papanduva	Santa Catarina	18,013	Sep 2017	68	48
Passagem Franca	Maranhão	17,296	Apr 2018	66	50
Pimenta	Minas Gerais	8,236	Dec 2018	31	6

*IBGE: Brazilian Institute of Geography and Statistics.*

Table 2 shows the number of sequenced ICs and relatives per region and their genotype regarding the presence of pathogenic or likely pathogenic variants (positive), no pathogenic variants (negative) or presence of a variant of uncertain significance (VUS), as well as the number of new cases derived from each enrolled IC.

**Table 2:** – ICs and relatives collected per region and their genotypes for the presence of FH genetic variants

Origin	ICs			Relatives			Number of relatives per identified ICs	Number of genotyped individuals per city
	Negative	Positive	VUS	Negative	Positive	VUS		
Bambuí	0	1	0	0	1	0	1	2
Bom Despacho	15	11	2	34	31	3	2.4	96
Buriti Bravo	4	0	0	0	0	0	0	4
Colinas	6	1	1	1	3	0	0.5	12
Lagoa do Mato	3	2	0	25	30	0	11	60
Luz	21	4	1	0	2	0	0.08	28
Major Vieira	1	3	0	48	44	0	23	96
Moema	1	4	0	36	32	0	13.6	73
Papanduva	4	2	1	50	46	0	13.7	103
Passagem Franca	3	5	0	55	45	0	12.5	108
Pimenta	6	2	1	0	4	0	0.4	13
Total	64	35	6	249	238	3	4.7	595

*IC: index case; VUS: variant of uncertain significance; FH: Familial hypercholesterolemia.*

Table 3 shows the three IC groups (negative, positive, or VUS) and their clinical and biochemical data. In total, 105 ICs were sequenced, and pathogenic or likely pathogenic variants were found in 36 (37.8%) individuals, and VUS in 5 (5.25%). Most ICs were female (67.6%), and when the clinical and biochemical characteristics were evaluated among the three groups, there was, as expected, a statistically significant difference regarding baseline (untreated) total cholesterol and LDL-C, with the positive group presenting the highest values of total cholesterol and LDL-C, 382 ± 150 mg/dL and 287 ± 148 mg/dL, respectively. Table 4 shows the clinical and biochemical characteristics of relatives.

**Table 3:** – Clinical and biochemical characteristics of negative, positive, and VUS-altered ICs

	Negative IC	(64)	Positive IC	(36)	IC VUS	(5)	p value
Females %	45 (70.3)	64	21 (58.3)	36	5 (100)	5	0.134
Males %	19 (29.7)	64	15 (41.7)	36	-	5	
Age (years)	54±15	64	44±19	36	56±16	5	0.015
Use of lipid lowering drugs	32 (50.0)	64	24 (66.7)	36	3 (60.0)	5	0.261
Early CAD	2 (3.1)	64	4 (11.1)	36	-	5	0.297
Xanthomas	3 (4.7)	64	3 (8.3)	36	1 (20.0)	5	0.365
Xanthelasmas	4 (6.3)	64	1 (2.8)	36	-	5	0.696
Corneal arcus	2 (3.1)	64	3 (8.3)	36	-	5	0.345
Current TC	279±65	62	316±107	36	302±28	5	0.102
Current LDL-C	195±56	64	234±104	36	207±35	5	0.051
Baseline TC	322±33	60	382±150	32	305±43	5	0.008
Baseline LDL-C	233±24	59	287±148	34	229±20	4	0.022

*CAD: coronary artery disease; IC: index case; LDL-C: low-density lipoprotein cholesterol; TC: total cholesterol; VUS: variant of uncertain significance. Early CAD defined as atherosclerotic cardiovascular disease event < 55 and 60 years of age in males and females, respectively; lipids in mg/dL; baseline lipids = untreated.*

**Table 4:** – Clinical and biochemical characteristics of negative and positive relatives

	Negative relatives	N (249)	Positive relatives	N (240)	p value
Females %	136 (54.6)	249	135 (56.3)	240	0.504
Males %	113 (45.4)	249	105 (43.8)	240	
Age (years)	40±21	249	38±21	240	0.710
In use of lipid lowering drugs	31 (12.4)	249	93 (38.8)	240	0.001
Early CAD	2 (0.8)	249	9 (3.8)	240	0.034
Xanthomas	6 (2.4)	249	17 (7.1)	240	0.013
Xanthelasmas	11 (4.4)	249	34 (14.2)	240	0.001
Corneal arcus	1 (0.4)	249	9 (3.8)	240	0.009
Current TC	198±51	114	309±86	127	0.001
Current LDL-C	124±42	192	233±75	198	0.001
Baseline TC	220±191	97	318±97	130	0.001
Baseline LDL-C	126±41	169	243±82	178	0.001

*CAD: coronary artery disease; LDL-C: low-density lipoprotein cholesterol; TC: total cholesterol. Early CAD defined as atherosclerotic cardiovascular disease event < 55 and 60 years of age in males and females, respectively; lipids in mg/dL; baseline lipids = untreated.*

Figure 2 shows the geographic distribution of the 11 cities located in 3 Brazilian states, the number of registered cases, the number of individuals genotyped, and the number of individuals with a pathogenic variant.

**Figure 2:** – Geographical distribution of cases, number of genotyped individuals, and number of individuals with an identified pathogenic variant (positive).

### Brazilian states, from top to bottom: Maranhão, Minas Gerais, and Santa Catarina

Table 5 shows all the encountered variants and the location where they were identified. In total, 21 different variants were identified with 3 variants appearing more frequently. Observed frequencies for these 3 variants suggest that they have founder effects in these localities. Six homozygous patients and one compound heterozygous in trans were found.

**Table 5:** – FH pathogenic variants, likely pathogenic variants and VUS found per city

Gene	Variant	Variant Classification	Bambuí	Bom Despacho	Luz	Pimenta	Moema	Buriti Bravo	Colinas	Lagoa do Mato	Passagem Franca	Major Vieira	Papanduva	Total
LDLR	Duplication from exon 4 to 8 (b)	Pathogenic	0	0	0	0	0	0	0	0	0	45^b^	41	86
LDLR	Duplication from promoter to exon 6	Pathogenic	0	0	0	0	0	1	4	29	49^a^	0	0	83
LDLR	p.Asp224Asn	Pathogenic	0	39	4	0	34	0	0	0	0	0	0	77
LDLR	p.Cys222*	Pathogenic	0	0	0	0	0	0	0	0	0	0	5	5
LDLR	c.1359-1G >C	Pathogenic	0	0	0	5	0	0	0	0	0	0	0	5
LDLR	p.Gly592Glu	Pathogenic	0	0	0	0	0	0	0	0	0	2	0	2
LDLR	p.Ala771Val	Pathogenic	0	0	1	0	0	0	0	0	0	0	0	1
LDLR	p.Pro699Leu	Pathogenic	0	0	1	0	0	0	0	0	0	0	0	1
LDLR	p.Asp601His	Likely Pathogenic	2	0	0	0	2	0	0	0	0	0	0	4
LDLR	p.Cys34Arg	Likely Pathogenic	0	1	0	0	0	0	0	0	0	0	0	1
LDLR	p.Arg257Trp	Likely Pathogenic	0	0	0	0	0	0	0	0	0	0	1	1
LDLR	p.Ser854Gly	Likely Pathogenic	0	2	0	0	0	0	0	0	0	0	0	2
LDLR	c.-228G>C	VUS	0	0	0	0	0	0	1	0	0	0	0	1
LDLR	p.Ala30Gly	VUS	0	0	0	1	0	0	0	0	0	0	0	1
APOB	p.Ala2790Thr	VUS	0	0	0	0	0	0	0	0	0	0	1	1
APOB	p.Met499Val	VUS	0	1	0	0	0	0	0	0	0	0	0	1
PCSK9	p.Arg237Trp	VUS	0	4	0	0	0	0	0	0	0	0	0	4
PCSK9	p.Arg357Cys	VUS	0	0	1	0	0	0	0	0	0	0	0	1
STAP1	p.Pro176Ser	VUS	0	0	0	1	0	0	0	0	0	0	0	1
LDLR	p.Cys222*	Pathogenic	0	0	0	0	0	0	0	0	0	0	1^c^	1^c^
LDLR	Duplication from exon 4 to 8	Pathogenic												
PCSK9	p.Arg215Cys	Likely Pathogenic	0	0	0	0	0	0	0	1	0	0	0	1^c^
APOB	p.Asp2213Asn	VUS												
APOB	p.Val3290Ile	VUS												
PCSK9	p.Arg215Cys	Likely Pathogenic	0	0	0	0	0	0	0	1	0	0	0	1^c^
APOB	p.Val3293lle	VUS												
PCSK9	p.Arg215Cys	Likely Pathogenic	0	0	0	0	0	0	0	1	0	0	0	1^c^
APOB	p.Asp2213Asn	VUS												

*2 homozygotes (b) 4 homozygotes (c) compound heterozygous in trans. VUS: variant of uncertain significance ; FH: Familial hypercholesterolemia.*

## Discussion

This study describes the results of the implementation of a cascade screening system for FH in 11 small Brazilian cities.

Despite the known cost benefits of cascade screening for FH, worldwide implementation has been suboptimal. Different local barriers and implementation hurdles have to be identified and overcome. How to implement cascade screening in small localities, for example, has been mainly overlooked. This challenge is greater in a continent-sized country like Brazil, where, in addition to the enormous geographic distances, there is inequality in access to health services. We have described the experience of HipercolBrasil in conducting comprehensive cascade screening in small towns in Brazil. In this new model, cascade genetic screening was carried out in cities that showed evidence of a higher prevalence of FH due to previous finding of individuals with the homozygous phenotype from the same city, or because those regions had reported elevated frequency of myocardial infarction.

Cities that had evidence of a founder effect were the ones that presented a higher identification of individuals affected per each IC analyzed (in descending order Major Vieira, Papanduva, Lagoa do Mato, and Passagem Franca). In these cities, we started from homozygous individuals whose parents were non-related and were born in different geographic regions. Clearly, whenever this situation is flagged by a cascade screening program, it deserves the deployment of a city-wide approach, because the costs-benefits of this scenario are the most advantageous. Implementing the genetic cascade in small towns proved to be more efficient when compared to the genetic cascade performed by Hipercol Brasil^11^ considering that the rates of family members per IC were 4.7 and 1.6, respectively (p < 0.0001).

It is important that the rate of tested family members per IC was also higher in cities with suspected founder effects. This probably occurred because these cities had a small number of inhabitants, and most relatives had some degree of familial relation. This did not occur in Bom Despacho, a city considerably larger than the others (45,624 inhabitants), and, although the number of family members collected was similar to that of other cities, there was a higher number of ICs collected (28) decreasing the rate of relatives/IC to 2,4. This situation exemplifies the tenuous equilibrium between city size and the success of the described approach.

Visited cities that were geographically close to cities with suspected founder effects (Bambuí, Buriti Bravo, Colinas, Pimenta, and Luz) had a low uptake of ICs and, consequently, a low number of identified relatives. This suggests that concentrating efforts in the selected municipality, as opposed to extending the approach to nearby towns, should be prioritized, and the capture of nearby potential cases should be left to the usual cascade screening mechanism.

## Conclusion

Cascade screening in small cities (fewer than 60,000 inhabitants) with a founder effect proved to be effective. However, some points might be of great importance in order for the cascade screening to be effective, and the following might be considered before deciding which cities to track: establishment of a formal partnership and explicit interest on the part of the local health department in receiving the program and performing the cascade screening; availability of clinical analysis laboratory datasets to carry out a retrospective survey of cholesterol tests; and dissemination via radio stations and social media regarding the disease and the program for greater adherence by the inhabitants.

This study is limited by the relative number of cities evaluated considering the continental size of Brazil. However, it suggests that the designed approach may be useful for detecting individuals with FH. In conclusion, our data suggest that, once detected, specific geographical regions warrant a targeted approach for the identification of clusters of FH individuals.
